# emPAI‐assisted strategy enhances screening and assessment of *Mycobacterium tuberculosis* infection serological markers

**DOI:** 10.1111/1751-7915.13829

**Published:** 2021-06-26

**Authors:** Guorong Ma, Pei Wang, Yanhui Yang, Wei Wang, Jinhua Ma, Lin Zhou, Junlin Ouyang, Rongxiu Li, Shulin Zhang

**Affiliations:** ^1^ School of Basic Medical Sciences Ningxia Medical University Yinchuan 750004 China; ^2^ Ningxia Key Laboratory of Prevention and Control of Common Infectious Diseases Ningxia Medical University Yinchuan 750004 China; ^3^ College of Biological Science and Engineering Northern University for Nationalities Yinchuan 750021 China; ^4^ State Key Laboratory of Microbial Metabolism School of Life Sciences & Biotechnology Shanghai Jiao Tong University Shanghai 200240 China; ^5^ Department of Immunology and Microbiology School of Medicine Shanghai Jiao Tong University Shanghai 200025 China; ^6^ Tuberculosis Research Center School of Medicine Shanghai Public Health Clinical Center Shanghai 201508 China

## Abstract

Discovering new serological markers of *Mycobacterium tuberculosis* (MTB) infection and establishing a rapid and efficient detection technology is of great significance for the prevention and control of tuberculosis. In this study, we established an exponentially modified protein abundance index (emPAI) value‐assisted strategy to investigate and improve the screening efficiency of serological biomarkers of tuberculosis. First, we used LC‐MS/MS to analyse MTB culture filtrate proteins (MTB‐CFPs), and 632 MTB proteins were identified. Then, the characteristic values of MTB‐CFPs – including emPAI value, molecular weight (Mw), isoelectric point (pI), grand average of hydropathy (GRAVY), transmembrane domain (TMD) and functional groups were calculated. Next, we successfully prepared 10 MTB proteins with emPAI value > 1.0 and recombinantly expressed these proteins in *Escherichia coli*. At the same time, 3 MTB proteins with emPAI between 0.1 and 0.5 were randomly selected as the control groups, and the immunogenicity of the recombinant MTB proteins was detected using ELISA. The sensitivity and receiver operating characteristic (ROC) curves were calculated for each recombinant MTB protein. The results showed that the areas under the curve (AUC) value of Rv2031c, Rv0577, Rv0831c, Rv0934 and Rv3248c were all higher than those of Rv3875 (AUC, 0.6643). Further analysis of the relationship between emPAI value and antibody sensitivity, AUC value and antibody affinity in mice immunized with recombinant MTB protein showed that emPAI values were positively correlated with them, and R‐squared value ranged from 0.64 to 0.79. The only exception was ESAT‐6 (encoded by the Rv3875 gene), which AUC value was relatively low owing to its strong immunosuppressive properties. This study provides a rationale for the serological marker screening of emPAI‐assisted tuberculosis clinical test. The results also provide new technical support for the screening of candidate serological markers of infectious diseases in the future.

## Graphical abstract

The areas under the curve (AUC) value of Rv2031c, Rv0577, Rv0831c, Rv0934, and Rv3248c were all higher than those of Rv3875 (AUC, 0.6643). Further analysis of the relationship between emPAI value and antibody sensitivity, AUC value, and antibody affinity in mice immunized with recombinant MTB‐protein showed that emPAI values were positively correlated with them, and R squared value ranged from 0.64 to 0.79. This study provides a rationale for the serological marker screening of emPAI‐assisted tuberculosis clinical test. The results also provide new technical support for the screening of candidate serological markers of infectious diseases in the future.

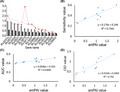

## Introduction

*Mycobacterium tuberculosis* (MTB) is the causative bacterium of tuberculosis. It is estimated that approximately 4 billion people worldwide have been latently infected, and about 10 million have developed tuberculosis. However, with timely diagnosis and the use of first‐line drugs for anti‐tuberculosis, the vast majority of patients can be cured. Therefore, rapid, inexpensive and simple testing methods and biomarkers are urgently needed (Annabel Baddeley *et al*., 2019). Especially in countries with high tuberculosis burden, such as India, China, Malaysia and Pakistan, where the number of patients is huge.

Active tuberculosis confirmation in clinics usually combines the typical tuberculosis symptoms, chest examinations with X‐ray or CT and microbial test evidence, including smear microscopy on sputum samples or MTB infection immunity tests, such as tuberculin skin test and antigen‐specific cytokines by ELISPOT (Robert S Wallis *et al*., 2013). The culture of sputum samples from patients has the following limitations. Firstly, it is prone to false negative with the low number of MTB in sputum. Secondly, the improper collection and storage of sputum samples may lead to infection of contact persons (Maciel *et al*., 2009). Thirdly, sputum culture is time‐consuming and takes 4–8 weeks (Abebe *et al*., 2007). The MTB antigen‐specific ELISPOT is a promising technique for tuberculosis detection in immune‐compromised patients; however, it lacks sufficient diagnostic power to distinguish patients from latently infected individuals and requires specially trained technicians (Sester *et al*., 2011). Moreover, antigen‐specific humoral immunogenicity can be easily applied with serum‐based colloidal gold test strips, enzyme‐linked immune‐sorbent assay (ELISA) testing (Weldingh *et al*., 2005), proteomic microarrays (Deng *et al*., 2014) and semi‐quantitative Western blotting (Liu *et al*., 2014). Despite the advantages of serological diagnostic methods, commercial serum‐antibody detection kits have not been effective in improving the sensitivity and specificity of tuberculosis clinical testing (Awaidy *et al*., 2011). The validity of this method is controversial, which spurred efforts to identify new serum markers of tuberculosis (You *et al*., 2017; Zhou *et al*., 2017; Ren *et al*., 2018).

The culture filtrate proteins of *M. tuberculosis* (MTB‐CFPs) play crucial roles in *M. tuberculosis* infections, bacterial survival and tuberculosis development, making them an attractive source of candidate biomarkers for diagnostic and vaccine antigens. Mass spectrometry (MS) analysis of the MTB‐CFPs detected 257–1176 proteins (Målen *et al*., 2007; Albrethsen *et al*., 2013). Most of them were low in abundance according to the MS data. In the immunogenic complex, the dose of antigen is too low to induce an immune response, leading to low immunologic tolerance (Abbas *et al*., 2001). Thus, we speculated that the highly abundant proteins in complex mixtures tend to incite stronger immune responses than the less abundant proteins.

The MS parameters, such as the number of hit matches, cover rates, and the number of peptides per protein was semi‐quantitative indicators for the abundance of identifications(Old *et al*., 2005), and the emPAI value in LC‐MS/MS analysis has been proven to be linear with the actual protein concentration (Ishihama *et al*., 2005). However, how to take advantage of the emPAI value in LC‐MS/MS analysis to speed up the discovery of the serological diagnostic values of the culture filtrate proteins remains unclear.

In this study, we assessed the serological diagnostic value of some MTB‐CFPs and found that the immunogenicity of most of the proteins was positively correlated with the emPAI value of MS analysis. Briefly, the composition of the MTB‐CFPs was analysed using LC‐MS/MS. The identifications have calculated the characteristics, including emPAI and functional groups. Then, the immunogenicity of the selected MTB proteins was measured using ELISA of serum from immunized mice and tuberculosis patients, and the sensitivity and specificity, as well as the receiver operating characteristic (ROC), of each MTB protein‐specific antibody level. Our study showed that the serological diagnostic value of the selected MTB‐CFPs increased with increasing emPAI value, except for the protein ESAT‐6 (encoded by the Rv3875 gene); therefore, emPAI value‐assisted strategies could enhance the discovery of serological markers in the development of clinical assays for tuberculosis.

## Results and Discussion

Proteomic LC‐MS approaches allow the identification of thousands of proteins from complex mixtures of MTB‐CFPs. However, it would be time‐consuming and expensive to extensively assess all proteins as biomarkers for the quick detection of tuberculosis. Therefore, it is crucial to set up a strategy to reduce the work and efforts to evaluate such a large number of MTB‐CFPs.

**Fig. 1:**
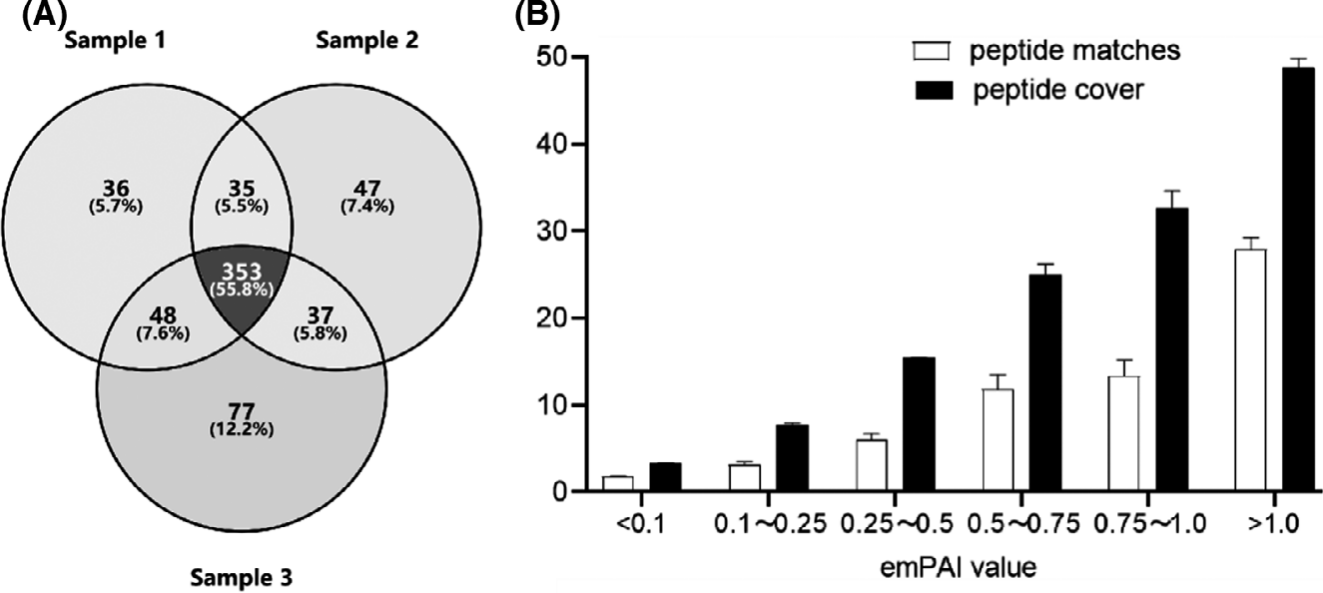
The number of identified MTB‐CFPs using MS analysis (A). The distribution with the peptide matches and peptide cover of identified proteins according to their emPAI value, of which, the peptide matches shown on the *Y*‐axis are counted in specific numbers, and the peptide cover is measured as a percentage of identifications against the total peptide chain residues (B).

LC‐MS/MS analysis of three batches of the MTB‐CFPs samples identified a total match hit of 632 proteins, with each MS run being 3732.7 ± 320.8 and identified 486 ± 25 (472, 472, and 515) proteins (Fig. 1A, Table S1.1). The detailed information of identifications in MTB‐CFPs by LC‐MS/MS has been shown in the supplementary materials (Table S2.1, Table S2.2 and Table S2.3). A total of 472 (74.7% of 632) proteins were detected at least two times, and 353 (55.8% of 632) proteins were detected three times (Fig. 1A). Thus, the MS analysis data for the MTB‐CFPs have high repeatability in this study.

### Biochemical characteristics of the identified MTB‐CFPs

The biochemical characteristics of the Mw, pI and GRAVY values of proteins are crucial for investigating the pathology and immunity of MTB‐CFPs, which can also serve as parameters for the selection of biomarker candidate proteins for recombinant expression in *Escherichia coli*. The Mw, pI, TMD and GRAVY values were calculated using the proteome discoverer software suite in the current study (Fig. S1).

The emPAI values of all the identified MTB‐CFPs were directly obtained by searching the Mascot database and filtered with a *p‐*value of less than 5% in this study. The identification distribution was as follows in six intervals of emPAI: <0.1, 0.1< and ≤0.25, 0.25< and ≤0.5, 0.5< and ≤0.75, 0.75< and ≤1, and ≥1, with each interval accounting for 19.3%, 34.5%, 21.4%, 9.5%,4.1% and 11.4%, respectively, of the total 632. Approximately 75.2% of the identifications were of emPAI < 0.5, which indicated that the overwhelming MTB‐CFPs were low‐abundance proteins. The average emPAI value (0.627) of three detection times using MS analysis was 3.6‐folds higher than the emPAI value (0.175) with one‐time detection (Table S1). Similarly, the peptide matches and protein coverage also increased proportionally as the emPAI value increased (Fig. 1B). The result indicates that proteins in the sample mixtures with higher emPAI values are more likely to be detected by MS.

### Analysis of the secretory pathway and functional group distribution of identifications

**Fig. 2:**
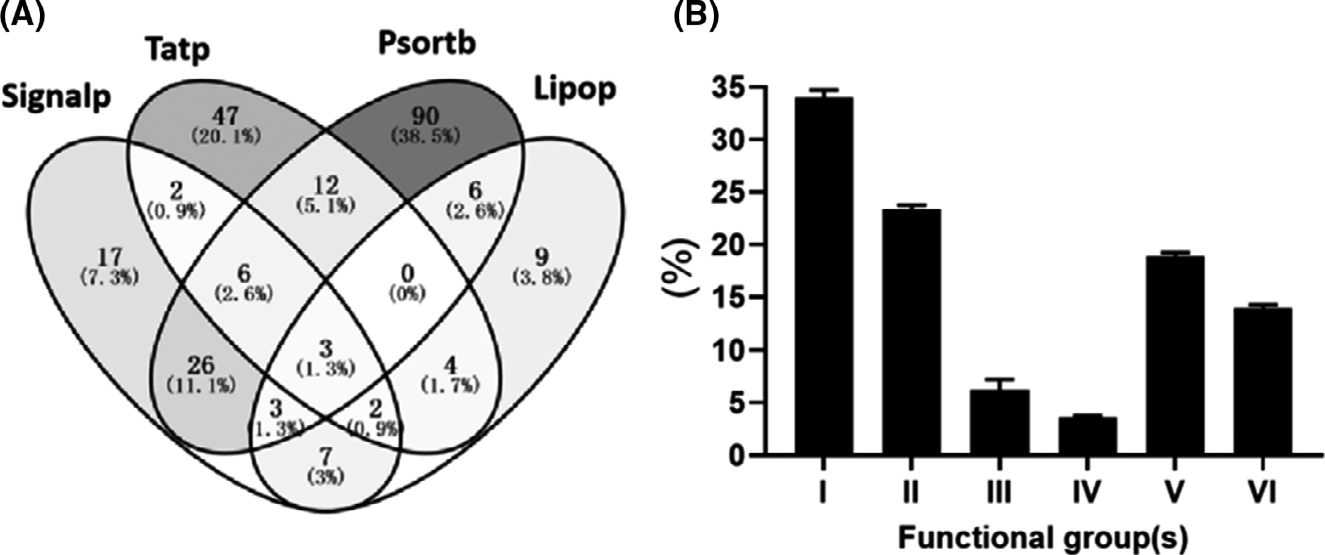
Venn diagram of the number of secreted proteins as determined with SignalP v3.0, PSORTb v3.0, LipoP v1.0, and TatP v1.0. (A). Functional groups of the detected proteins based on the TubercuList server, of which I denotes small‐molecule metabolism, II denotes macromolecule metabolism, III denotes cell processes of transport/binding proteins, chaperones, protein and peptide secretion, IV denotes virulence IS elements, repeated sequences, phage, and PE and PPE families, V denotes conserved hypothetical proteins and VI denotes undetermined proteins (B).

Similar to most bacteria, MTB secretes many proteins to the outside of the bacterium in more than eight different ways (Ligon, *et al*., 2012). Therefore, many MTB proteins were detected in culture filtrates. In this study, all the identified proteins were analysed using five bio‐informational strategies (Fig. 2A), and 234 (37.03% of 632) proteins had at least one of the following criteria: (i) the sequence of 66 proteins was recognized by signal peptidase I or II, (ii) the presence of 76 proteins belonging to the twin‐arginine tag (Tat) motif and (iii) the subcellular localization of 149 identifications was determined as exported proteins by Psortb v3.0 prediction. The subcellular location of 632 identified MTB‐CFPs was as follows: 127 in the cytoplasmic membrane, 372 in the cytoplasm, 14 in the cell wall, 18 in the extracellular compartment, and 115 in the multi‐compartments (Fig. 2A, Table S1). Consistent with previous proteomic studies, more than half of the MTB‐CFPs are cytoplasmic proteins. It was presumed that these multi‐location proteins are released into the culture media via multiple mechanisms.

Functional classification of the proteome of *M. tuberculosis* H_37_Rv fell into six distinct groups, which consisted of 84 sub‐groups (http://genolist.pasteur.fr/tuberculist/). The majority of MTB‐CFPs identified were involved in the metabolism of the small molecules and macromolecules. Interestingly, < 1/5 (~ 16%) identifications were the virulence, phage, and PE and PPE family proteins (Fig. 2B). These proteins play crucial roles in virulence. Approximately one‐third was conserved hypothetical proteins (V) and undetermined proteins (VI), suggesting more painstaking efforts are needed to elucidate their function in *M. tuberculosis* survival and pathology.

### Selection of proteins for immunogenicity assessment

Rapid and efficient acquisition of candidate proteins is crucial for the study of serum markers in tuberculosis diagnosis. However, the expression of the 632 detected MTB‐CFPs in *E. coli* using traditional methods and the evaluation of their immunogenicity using ELISA would have been laborious. Furthermore, it is extremely difficult to obtain membrane proteins, hydrophobic proteins and extra‐large proteins in *E. coli*. Additionally, the extra‐small‐size proteins and/or extreme pI are easily lost using SDS‐PAGE. Therefore, proteins with stable physiological and biochemical properties, easy to express in *E. coli* and purified were selected for further study.

**Fig. 3:**
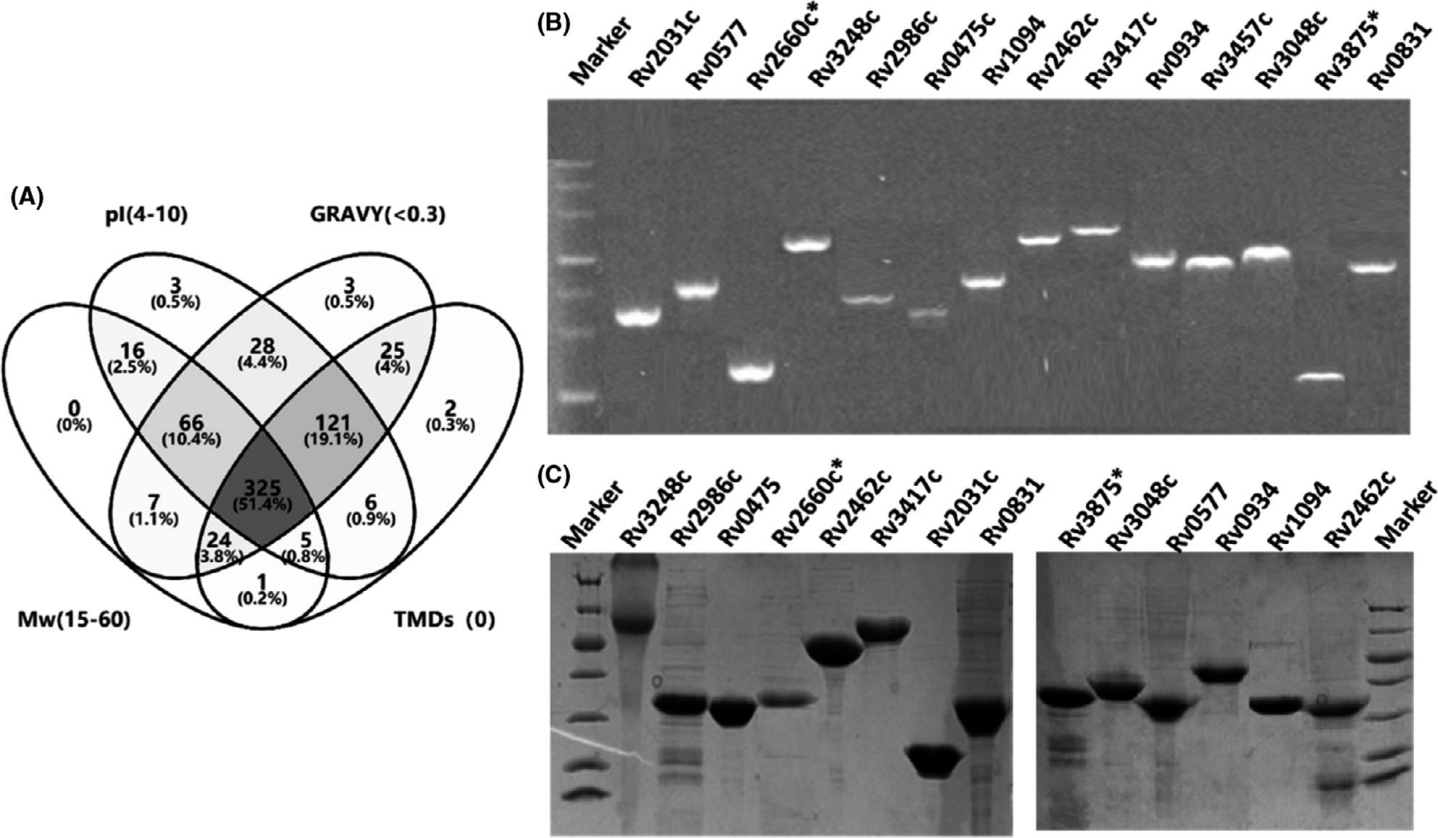
The candidate protein selection was based on the biochemical properties of Mw, pI, GRAVY and TMD (A). Each recombinant protein was listed by its *M. tuberculosis* H37Rv gene number, which was amplified via PCR and subsequently cloned into the pET28a or pET32a (*) expression vector (B). The recombinant MTB proteins were purified and separated using 12% SDS‐PAGE and Coomassie blue staining (C).

In summary, 345 proteins (51.4% of 632) with Mw of 15–60 kDa, pI of 4.0–10, Gravy < 0.3 and 0 transmembrane domains should be expressed (Fig. 3A). It is still a heavy task and burden to evaluate 345 proteins. Then, it is further shortlisted by peptides coverage of > 30%, in addition to emPAI value of > 1.0, Mw of 15–60 kDa, pI of 4.0–10, Gravy < 0.3 and 0 transmembrane domains, resulting in 27 candidates. Fifteen proteins have been reported and three of them (proteins encoded by the genes Rv0934, Rv2031c and Rv3875c), with good clinical sensitivity and specificity as the positive control in this study, were retained. The remaining 12 proteins were expressed by classical methods of genetic engineering. Meanwhile, three proteins with emPAI value between 0.05 and 0.5 were randomly selected from 287 identifications as the control group (Fig. 3B, Table S2). For primer design, the signal peptides of expression, such as PstsI (encoded by the gene Rv0934), were removed. Briefly, these proteins were expressed and purified according to methods described previously (Ma *et al*., 2017a). Some recombinant MTB proteins were expressed in a soluble stage, and included pET‐28a‐Rv0577, pET‐28a‐Rv2031c, pET‐32a‐Rv3875, pET‐28a‐Rv1094, pET‐32a‐Rv2660, pET‐28a‐Rv2986c and pET‐28a‐Rv3457, while others were expressed in inclusion bodies (Fig. 3B). In total, 10 proteins were obtained along with the three control proteins (Table S2).

### Evaluation of the immunogenicity in the serum of mice

**Fig. 4:**
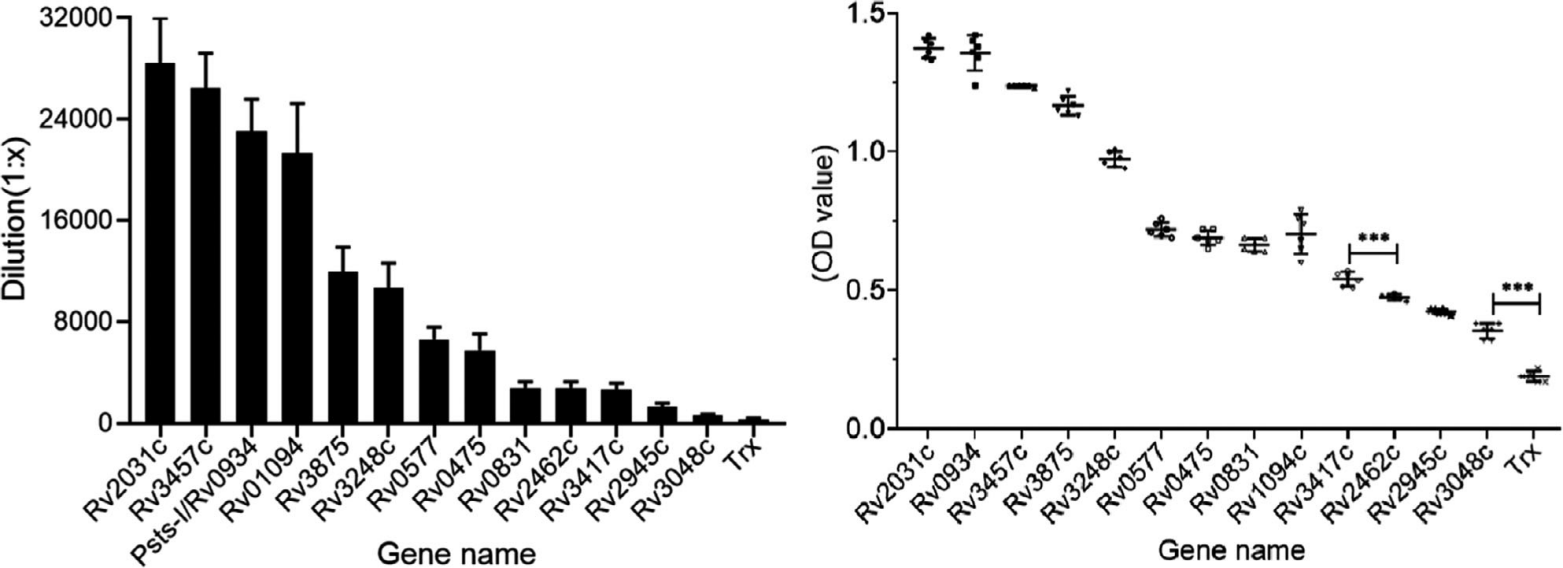
The titre of each recombinant‐MTB protein against the sera of mouse anti‐whole‐MTB‐CFPs (A). The value of absorbance (OD 450) of unfractionated MTB‐CFPs against the sera of mouse‐anti‐recombinant protein (B).

Humoral immunogenicity of the recombinant MTB proteins was calculated using murine anti‐whole‐MTB‐CFPs and anti‐recombinant‐protein sera respectively. The trx (tag of vector pET‐32a) protein was used as the negative control. It showed that 66.67% (6/9) of screening proteins (emPAI > 1.0) were recognized strongly by the murine‐anti‐MTB‐CFP serum, with antibody titres ranging from 1:51 200 to 1:12 800, and two proteins (emPAI > 1) with antibody titre of nearly 1:6400. In the control proteins, 2 out of 3 (emPAI between 0.01 and 0.5) were detected with an antibody titre of < 1:800 (Fig. 4A). Notably, the recombinant‐MTB protein encoded by the Rv2660c gene was detected with the lowest antibody titres, and this protein was not detected using LC‐MS in this study. The negative control Trx could not be recognized by the anti‐whole‐MTB‐CFPs serum (Fig. 4B). Therefore, we speculate that the antibody titre in the serum of the host against the antigen is proportional to the protein abundance observed in the MTB‐CFPs fraction.

Another factor that should also be considered is that the recombinant protein was not recognized by the anti‐whole‐MTB‐CFPs serum, indicating that this protein is either absent in the MTB‐CFPs fraction or is not recognized because of the poor activity of the recombinant MTB protein.

### Evaluation of the sensitivity and specificity in the serum of humans

We performed receiver operating characteristic (ROC) analysis of antibody responses of sera from patients with tuberculosis and healthy control individuals. The AUC values of 13 proteins were obtained (Table 1). The recombinant MTB protein Rv3875 was designated as the control. Compared with the AUC value of Rv3875 [AUC, 0.6643; 95% confidence interval (CI), 0.5725–0.7561], five recombinant‐MTB proteins, namely Rv2031c, Rv0577, Rv0831c, Rv0934 and Rv3248c had higher AUC values for serodiagnosis of tuberculosis patients. All of these proteins were identified with an emPAI value of > 1.0 in the culture filtrate of *M. tuberculosis*. These results indicate that these antigens are promising serological biomarkers for the diagnosis of tuberculosis. For the three control recombinant‐MTB proteins with a value of 0.1 < emPAI value < 0.5, their AUC value was significantly lower than that of the recombinant‐MTB protein with emPAI value of > 1.0.

**Table 1:** The 13 selected recombinant‐MTB proteins by serological initial screening.

Rv No.	emPAI	AUC^a^	95% CI	Protein description
Rv2031c	1.94	0.7781	0.7172–0.8390	heat shock protein Hsp20 family
Rv0577	1.09	0.7659	0.7073–0.8246	Conserved hypothetical protein Cfp30B
Rv0831c	1.02	0.7381	0.6593–0.8168	Conserved hypothetical protein
Rv0934	1.31	0.7206	0.6509–0.7903	Phosphate‐binding protein
Rv3248c	1.09	0.6736	0.5955–0.7517	*S*‐adenosyl‐l‐homocysteine hydrolase
Rv3875	2.97	0.6643	0.5725–0.7561	ESAT‐6‐like protein
Rv0475	1.06	0.6526	0.6012–0.7210	Heparin‐binding hemagglutinin
Rv2462c	0.50	0.646	0.5641–0.7279	chaperone, similar to trigger factor
Rv3457c	1.20	0.6361	0.5425–0.7296	DNA‐directed RNA polymerase subunit
Rv1094	0.45	0.6301	0.5332–0.7156	Acyl‐ACP desaturase
Rv2945c	0.14	0.6121	0.5225–0.7096	Lipoprotein LppX
Rv3048c	0.09	0.5841	0.5322–0.6349	Ribonucleotide‐diphosphate reductase
Rv2660c	a	0.5613	0.4825–0.6402	hypothetical protein

'a' denotes this protein was undetected in this study.

**Fig. 5:**
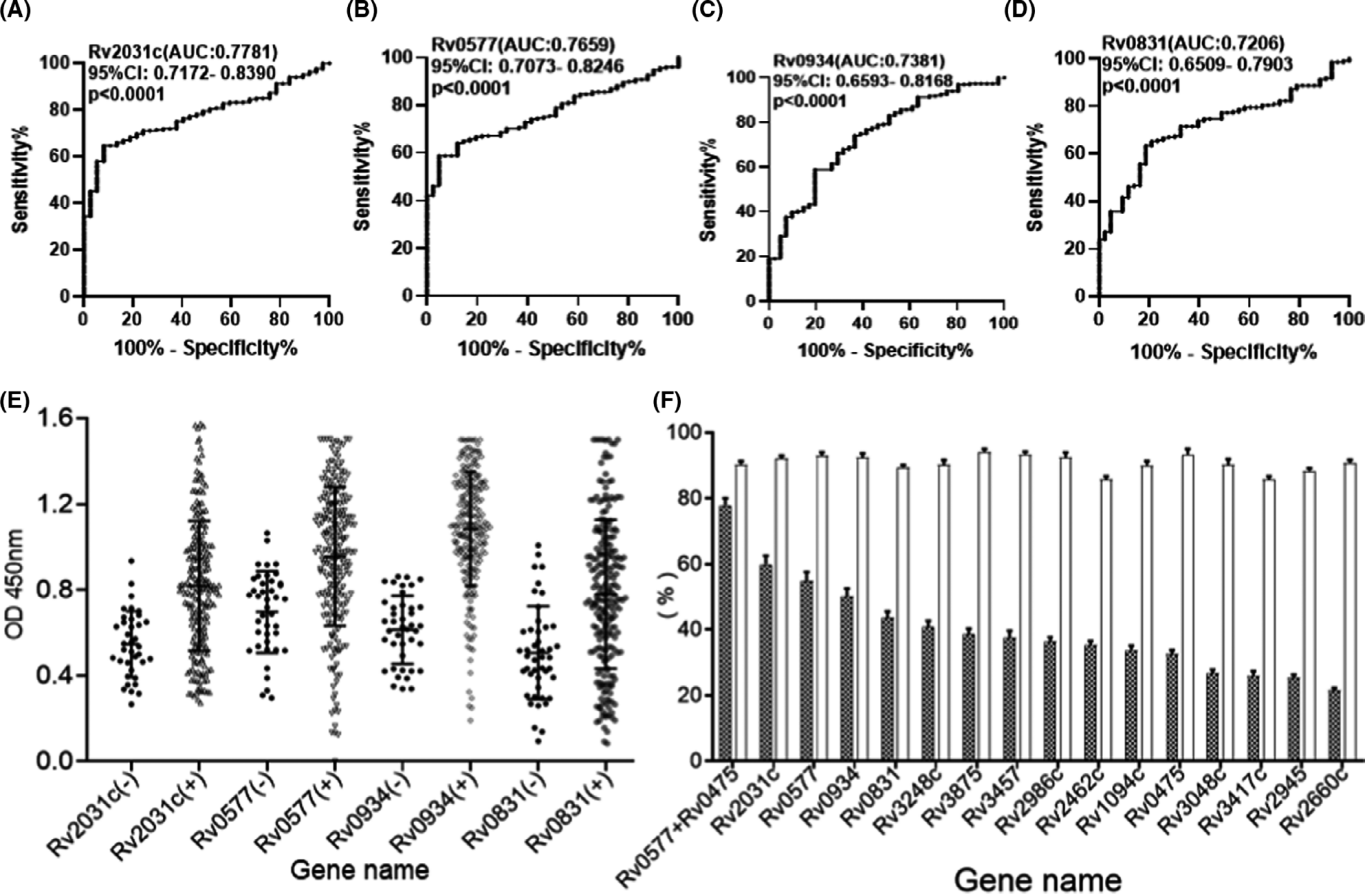
The levels of antibodies in the sera of tuberculosis patients and healthy control individuals against the recombinant MTB proteins were determined using the ELISA method. The sensitivity and specificity of these four proteins were determined using ROC curve analysis (A–D). The OD values of the recombinant MTB proteins pET28a‐Rv2031c, pET28a‐Rv0577，pET28a‐Rv0934 and pET28a‐Rv0831c (E). The sensitivity and specificity of the response of 13 recombinant‐MTB proteins to the serum of tuberculosis patients and healthy control individuals using ELISA. The grey bars represent the sensitivity of each recombinant protein, and the white bar represents the specificity (F).

To better evaluate and analyse the feasibility of these proteins based on emPAI values, their sensitivity and specificity were evaluated using an ELISA assay by screening sera samples from tuberculosis patients and healthy control individuals. The optical density at 450 nm (OD450) ratio of tuberculosis patient sera was more than the average OD value in healthy control individuals, plus three standard deviations determined to be positive. The sensitivity varied from 18.6% to 59.9% and the specificity ranged from 89.9% to 95.6% (Fig. 5F).

**Fig. 6:**
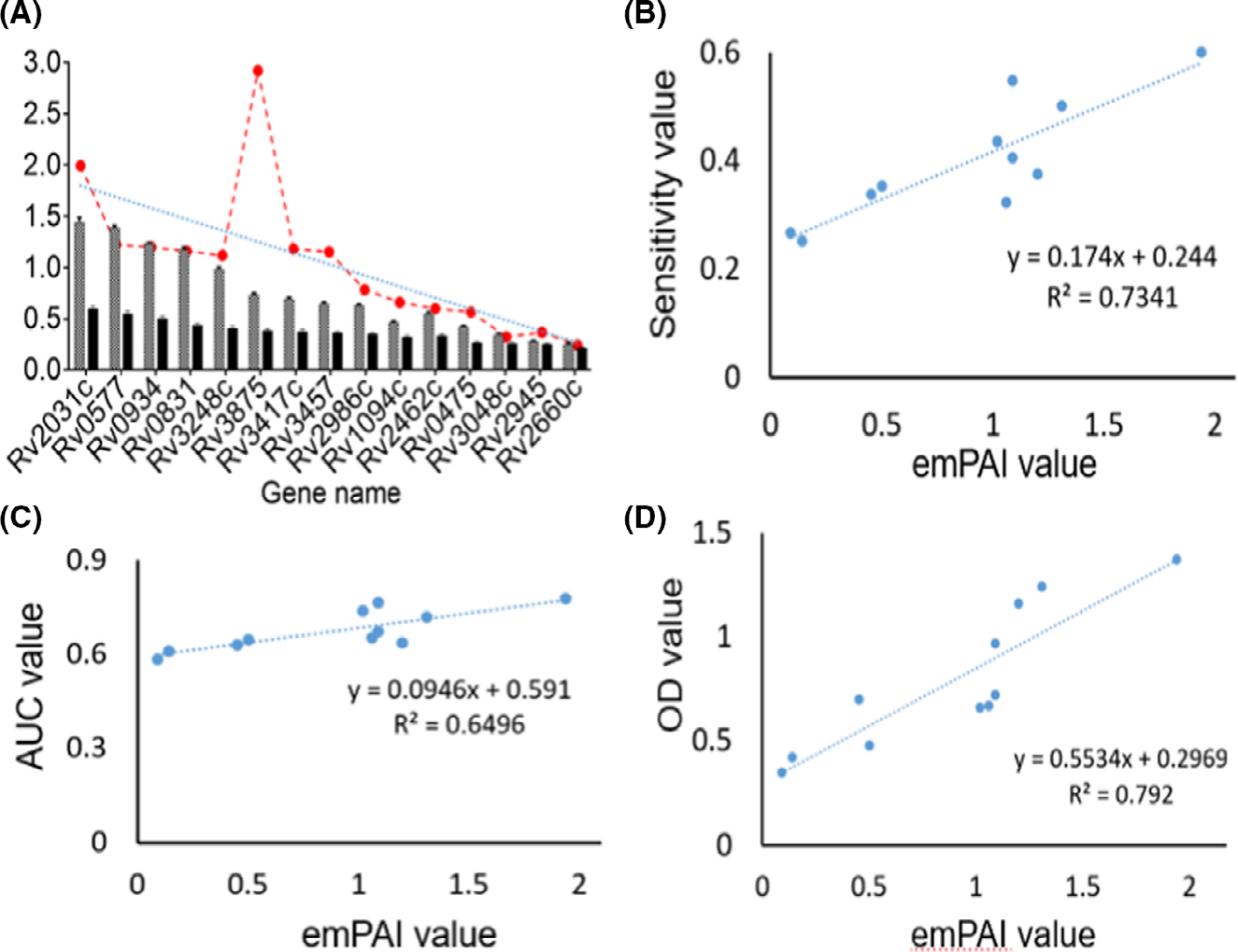
The correlation between the emPAI value of the selected *M. tuberculosis* proteins and the detected data of sensitivity, AUC and OD value. The blue dot line is the trend‐line between the emPAI values of the 13 selected MTB‐CFPs and the mouse titer OD value (black bar) and the human serum antibody sensitivity (grey bar). Each red dot represents the average emPAI value of the corresponding gene‐encoding protein in MTB‐CFPs (A). The correlation between the emPAI value of 11 selected proteins（excluding the proteins encoded by genes Rv3875 and Rv2660）and its clinical sensitivity, AUC value in sera of tuberculosis patients, and the OD value from immunogenicity of 11 selected‐protein‐immunized mice (B–D).

The emPAI values of 10 selected proteins and three control proteins correlated with the sensitivity of sera from the clinical evaluation of tuberculosis patients, the AUC value and the immune values of mice respectively (Fig. 6A). The R‐squared value is an indicator of the fitting degree of the trend line, and its numerical value can reflect the fitting degree between the estimated value of the trend line and the corresponding actual data. The higher is the fitting degree, the higher the reliability of the trend line. The R‐squared value ranges from 0 to 1 and is called the determining factor. When the R‐squared value of the trend line is equal to or close to 1, its reliability is the highest. Otherwise, its reliability is low.

By analysing the correlation between the emPAI value and antibody sensitivity, the emPAI values were revealed to positively correlate with the AUC value and antibody affinity of mice immunized with the corresponding recombinant MTB protein. The R‐squared values ranged from 0.64 to 0.79 (Fig. 6B–D). Among the above three groups of data, the highest correlation was the OD value of the antibody in mice after screening and recombining the expressed protein to immunize mice and the protein source solution of the culture filtrate of tuberculosis bacteria. The comparison fully reflects the abundance of the corresponding protein in the culture filtrate and the concentration of the corresponding antibody induced by the body. The reaction between the recombinant MTB protein and the corresponding antibody in the sera of the patients are affected by the amount of bacterium that infects the patient, the course of the disease and the physical condition of the patient. Therefore, the *R* ^2^ values of the selected protein and its OD value are higher than those of the patients. The sensitivity and AUC values were less than those of the former (Fig. 6B–D).

The serum antibody concentration of the protein ESAT‐6 encoded by the gene Rv3875 was relatively lower than that of the other selected proteins in this study, although this protein has a very high abundance in MTB‐CFPs. The protein ESAT‐6 has reduced NO and ROS production (Atefeh Seghatoleslam *et al*., 2016), regulates autophagous response through SOD‐2 (Yabaji *et al*., 2020) and interacts with beta‐2‐microglobulin (β2 M) reducing antigen presentation (Sreejit *et al*., 2014), thus suppressing macrophage immune response. The other mechanism we speculated included: (i) The antigens of very high levels of concentration are to induce high‐dose immunologic tolerance since the very highly abundant proteins will lead to activation of the regulatory T cells, thereby suppressing the immune response. (ii) The number of B cell epitopes on this protein is small. (iii) The molecular weight of this protein is only 6 kDa, and it is known that small proteins can easily cause immune tolerance. As a result, the correlation between emPAI value and the corresponding antibody concentration decreased.

Among the six proteins with higher AUC values in this study, the proteins encoded by Rv2031c, Rv0934 and Rv3875 were identified as proteins that inhibit the antimicrobial effects of macrophages, inorganic phosphate transport antigen and apoptogenic, and Esx‐1 component or substrate respectively. These results are consistent with the results of the functional classification in this study (Table S1.2). Rv0934 and Rv2031c are promising and effective markers for serological antibody detection (Ireton *et al*., 2010; Kunnath‐Velayudhan *et al*., 2010). The protein encoded by Rv3875 is one of the two core antigens for detecting tuberculosis patients based on the ELISPOT strategy (Philip *et al*., 2007; Sun *et al*., 2011). The physiological function and toxicological factors of proteins encoded by Rv0577 were responsible for neutral red staining of virulent strains of *M. tuberculosis* (Andreu *et al*., 2004); however, the proteins encoded by Rv0831c and Rv3248c are rarely reported. Data on its clinical evaluation as a tuberculosis test are also scarce. The functional groups of proteins encoded by genes Rv0577 and Rv0831c are the conserved hypotheticals (Table S1). The protein encoded by gene Rv3248c belongs to sulfur metabolism according to its functional classification (Table S1).

With technical development, more than one thousand proteins can be detected from protein mixtures by the MS approach. The emPAI value is an effective and promising parameter of label‐free and has a linear relationship with the logarithm of protein concentration in LC‐MS analysis. It is well known that a higher host immune response would be induced by the high concentration of exogenous proteins. Thus, the highly abundant proteins in the analyte were screened based on their emPAI value in this study. Bioinformatics was used to predict the properties of these identifications, select suitable expression proteins in *E. coli* and evaluate the immunogenicity, sensitivity and specificity of these recombinant MTB proteins. Finally, serum markers suitable for the treatment of tuberculosis clinical diseases were assessed.

Furthermore, it is known that patient antibody responses to MTB antigens are heterogeneous due to the stage of the disease, differences in HLA types, the strain of bacilli and the bacillary load. Therefore, the desired sensitivity of detection of antibodies with a single antigen is of limited diagnosis of tuberculosis. Thus, we compared and analysed the data of individual recombinant‐MTB proteins to identify the best combination that improves sensitivity and specificity. The principle for determining a negative result in the combination is that the same serum has a negative ELISA data for all antigens. For the positive result of the combination is any one of the antigens is positive in the same patient serum. As observed in earlier studies, we also found that the sensitivity increased, but the specificity was reduced after combining the diagnostic data of all the individuals. The best pair (or cocktail) of antigens produced a maximum specificity of 89.1–92.2% with Rv0577c and Rv0475, and a sensitivity of 75.3–82.1% (Fig. 6D). These results are encouraging, and it is now necessary to optimize the assays and to investigate more MTB‐CFPs for the development of better tuberculosis diagnostics.

To reduce the change in the original concentration with each MTB protein in culture filtrates using mass spectrometry analysis, the MTB‐CFPs were not fractionated in the current study. Some of the low abundant proteins were undetected by MS analysis, because their peak signals were masked by the highly abundant proteins.

The protein encoded by gene Rv2660c was used as the control in this study based on the following reasons. First, the gene Rv2660c belonged to region difference 13 (RD13) encoded by MTB, and it was closely related to dormancy of bacterial strain. Second, the mRNA of Rv2660c was significantly upregulated in culture of MTB under starvation in vitro, which was crucial for the bacterial strain to adapt to nutrient deficiency and hypoxia. Third, as one of the three component protein of the vaccine H56 (antigens Ag85B, ESAT‐6 and protein encoded by Rv2660c), it was evaluated as an important candidate protein in vaccine development. Meanwhile, the data of this study were also shown that the recombinant protein by Rv2660c was of low immunogenicity and did not protect the mice (Aagaard *et al*., 2011) . Fourth, this protein is not identified in the current study by MS analysis, and it was also not detected in MTB‐CFPs in previously studies (Målen *et al*., 2007, Albrethsen *et al*., 2013). Furthermore, the sensitivity and specificity of this protein in sera of tuberculosis patients have not been reported. Therefore, this protein was prepared, then the sensitivity and specificity of which were evaluated in sera of tuberculosis patients and the sera from MTB‐CFPs inoculated mice. The results were shown that the recombinant MTB protein encoded by Rv2660c had a low sensitivity in sera of tuberculosis patients and mice and MTB‐CFPs inoculated mice than that of the 10 high abundant recombinant proteins respectively. Therefore, we speculate that this protein is highly likely to belong to the low abundant protein in MTB, and it may be not a good serum marker for tuberculosis diagnosis.

In conclusion, the emPAI value is a promising criterion for the diagnosis of serum‐biomarker screening of tuberculosis. Except for these 13 selected proteins, other identifications with emPAI vale of > 0.5 are also worthy of investigation based on this method. In particular, the proteins that are predicted to be secreted, and low sequence similarity with the host proteome are worthy of future evaluation. Further investigation into these proteins is warranted for the development of novel biomarkers that can be used for the screening of bacillus infections in low‐ and middle‐income countries. Importantly, new serological biomarkers of tuberculosis will be selected by investigating the profile of cytoplasmic proteins of *M. tuberculosis* using a similar strategy in the present study.

## Experimental procedures

### Preparation and fractionation of MTB‐CFPs

Proteins from the culture filtrate of *M. tuberculosis* H_37_Rv (ATCC 27294) were prepared using a previously described method (Ma *et al*., 2017b). In brief, the activated bacteria were re‐suspended in Sauton’s media and incubated at 37°C without shaking for approximately 21 days, until a pellicle had fully developed. The concentration of MTB‐CFPs was maintained at 4 mg ml^‐1^ with 10 mM PBS (pH 7.4) via ultrafiltration using an Amicon Ultra‐3K filter unit (Merck Millipore, CORK, Ireland) and assayed using a Pierce BCA protein assay kit (Thermo Scientific, Waltham, MA, USA).

### Tryptic digestion

Three batches of MTB‐CFPs samples were prepared. 20 µl (30 μg) of the fraction was mixed with 5 µl 100 mM DTT, incubated at 37℃ for 3 h. Then, 5 µl 450 mM IAA was added and incubated at 37℃ for 30 min in the dark before addition of 1 µl trypsin (V5280; Mass Spectrometry Grade, Promega, MI, USA). The reaction was quenched after incubation at 37℃ for 16 h by addition of 3.5 µl formic acid. The resulting peptides were vacuum dried and re‐suspended in 0.1% formic acid.

### LC MS/MS analysis

Each fraction was analysed by an LC system (Dionex‐ultimate 3000 Nano LC; Thermo Scientific) coupled to an ESI‐Q‐TOF mass spectrometer (maxis, impact; Bruker Daltonik, Bremen, Germany) in data‐dependent acquisition mode (m/z 350–1500). The peptides were loaded onto a C 18 capillary column (75 μm × cm) and eluted at a constant flow rate of 400 nl min^‐1^ by a solvent mix consisting solvent A (0.1% formic acid in water) and solvent B (0.1% formic acid in acetonitrile) in a multi‐step gradient scheme: 2–30% B in 87 min, 30–50% B in 10 min, 50–80% B in 10 min and 80%B hold for 10 min. The mass spectrometer was set as one full MS scan followed by 10 MS/MS scans on the 10 most intense ions from the MS spectrum. The Source Capillary was set at 1900v, and the flow and temperature of dry gas was 2.0 l min^‐1^ and 120°C respectively.

### Protein identification

Tandem mass spectra were extracted, and charge state convoluted and deisotoped by Compass Data Analysis (version 4.1; Bruker Daltonics). The peak list was directly generated from raw data using centroid algorithm with peak width set as 0.1 m/z and intensity above 100. No peak smoothing was performed, or any filter applied. After the charge states were calculated, the deisotoped peak list was exported as an mgf file. Mascot (version 2.4; Matrix Science, London, UK) was initiated to search *Mycobacterium tuberculosis* H37Rv (4875 entries) using the Uniprot TREMBLE database with the following parameters: carbamidomethyls of cysteine as fixed modifications, oxidation of methionine as variable modifications, trypsin specificity with two missed cleavages allowed, mass tolerance 20 ppm for MS precursors and 0.05 Da for fragment ions, peptide charges +2, +3 and +4. Peptide identification was established according to the MASCOT score (*P* ≤ 0.05).

### Bioinformatics analysis

*M. tuberculosis* belongs to the gram‐positive family of bacteria and possesses at least seven different secretory pathways (Ligon *et al*., 2012).

As in our previous studies, all the detected proteins were systematically analysed using the bioinformatics methods of SignalP v3.0, TatP v1.0, TMHMM v2.0, Lipop v1.0 and PSORTb v3.0 (Ma *et al*., 2017b). Functional classifications were performed on the TubercuList server (Albrethsen *et al*., 2013). The theoretical Mw, pI and emPAI values were calculated using the Proteome Discoverer software.

The emPAI value calculation:

(1) $$\text{emPAI}=10{(}^{\text{Nobsd}/\text{Nobsbl}})‐1,$$

where Nobsd and Nobsbl are the number of observed peptides per protein and the number of observable peptides per protein respectively (Ishihama *et al*., 2005; Shinoda, *et al*., 2010).

### Selection of proteins and expression

The expression and purification of each identified MTB‐CFP were necessary for the investigation of the biomarker. However, the expression success rate of MTB‐CFPs in *the E. coli* system was less than 50% owing to their high GC content and different codon usage. In this study, the identifications were shortlisted by characteristics: (I) highly abundant in CFPs with emPAI value of > 1.0 and peptide cover rate of > 30% in MS analysis, (II) easy expression in *E. coli*, and biochemical properties of 15 kDa < Mw < 60 kDa, 4 < pI < 10, GRAVY < 0.3, and without the transmembrane domains. Meanwhile, three identifications with emPAI value between 0.01 and 0.5 were randomly selected and prepared as the control group.

The recombinant plasmid of the selected proteins was constructed using a previously described method (Ma et al., 2017a). Insoluble proteins were refolded by dialysis to decrease the urea concentration from 8–2 M. All the recombinant MTB proteins contained a His‐tag and were initially purified using nickel Sepharose 6FF resin, and further purified using molecular‐exclusion column chromatography on Superdex^TM^ 75 (GE, Healthcare, Boston, MA, USA), according to the recommended protocol.

### Murine anti‐sera preparation

#### Vaccination of murine

A total of 120 Balb/c mice (female, 8 weeks) were purchased from SLAC Laboratory Animal Inc. (SLAC, Shanghai, China), divided equally into 20 groups and housed under specific pathogen‐free conditions. The animal experiments were performed in accordance with the guidelines of the Chinese Council on Animal Care. In the negative control group, six mice were immunized with a 50 µl mixture of 10 mM PBS and Freund's incomplete adjuvant (Sigma‐Aldrich Co. LLC, Louis, MI, USA). In the 13 recombinant MTB proteins, the mice were vaccinated with a mixture of the equivalent volume of purified recombinant protein (2 mg ml^‐1^) and Freund's incomplete adjuvant. Each mouse was vaccinated every two weeks, with 100, 50 and 50 µg antigens. The same method was used to prepare mouse anti‐whole‐MTB‐CFPs and mouse anti‐recombinant‐MTB protein sera respectively.

#### Detection of anti‐sera titre

To investigate the immunogenicity of the whole fraction of MTB, the antibody titre was detected using ELISA. The optimal concentration of each purified recombinant MTB protein, the whole serum and rabbit anti‐mouse IgG‐HRP was determined following an orthogonal test. The final concentrations were 0.02–0.1 µg ml^‐1^, 1:100–1: 200 (v/v) and 1:10 000–1:15000 (v/v) dilutions respectively.

### Sensitivity and specificity of the recombinant MTB proteins

#### Serum collection

Serum samples from 242 patients with tuberculosis and 45 healthy controls were recruited from the Shanghai Pulmonary Hospital, China (Table 2). Informed consent was obtained from each participant.

**Table 2:** The detail information of sera with tuberculosis (TB) patients and healthy control individuals (HCS).

Groups	TB group‐1	TB group‐2	TB group‐3	Healthy
Number of the samples	97	88	57	45
Meaning age (years)	54.4	57.6	61.9	37.3
Range of age (years)	17–82	19–71	21–74	14–71
Gender (male/female)	55/42	49/39	32/25	27/18
Acid‐fast bacteria staining	44	35	28	a
Sputum culture	72	62	44	a
X‐ray or CT	54	47	29	a
Secondary tuberculosis	61	50	49	a
Tuberculous pleurisy	18	16	10	a

‘a’ denoted no clinical test data available.

TB group‐1, TB group‐2 and TB group‐3 denote the sera of tuberculosis patients were collected in the hospital by three times.

#### Serological detection

The purified recombinant MTB protein (0.02–0.1. µg ml^‐1^) was coated onto microplate wells and incubated with 100 µL patient sera (1:20 dilution in 5% skimmed milk‐PBS) at 37°C for 1 h. The plates were washed with PBST, and 100 µl of HRP‐conjugated rabbit anti‐human immunoglobulin (IgG) antibody (1:10 000 dilution) was added. After washing with PBST, 100 µl (0.1 mg ml^‐1^) tetramethylbenzidine peroxidase substrate was added, and the reaction was allowed to proceed for 10–30 min prior to the addition of 50 µl of 2 M H_2_SO_4_. The optical density (OD) was measured at 450 nm using an EnSpire 2300 (PerkinElmer, Waltham, MA, USA).

### Statistical analysis

Each serum sample was assayed in triplicates. The positive cut‐off for serum was also determined by the OD value, which was larger than the average OD value in the sera from healthy control individuals, plus three standard deviations thereof (Weldingh *et al*., 2005). The sensitivity and specificity of each serum of humans against the recombinant MTB protein were evaluated by an ROC curve using GraphPad Prism V8 (GraphPad Software, San Diego, CA, USA).

In statistics, R‐squared is calculated as follows:

$$\begin{array}{ll} R^{2}\;= & \;\text{sum}\;\text{of}\;\text{regressed}\;\text{squares}\;\left(\text{SSREg}\right)\\ & \text{/total}\;\text{sum}\;\text{of}\;\text{squares}\;\text{(sstotal),} \end{array}$$

where the sum of regressed squares = total sum of squares ‐ residual sum of squares (SSresid).

## Funding Information

The National Science & Technology Major Projects of China (Major infectious Diseases 2017ZX10201301‐003‐004), This work was supported by Ningxia hui autonomous region key research and development (talent introduction project: 2018BEB04033), Ningxia hui autonomous region education department outstanding young teachers training fund project (NGY2018‐79), The Ningxia hui autonomous region key R&D (general project 2019BEG03065), Superior discipline group (Orthopedics) construction research project in 2019 of Ningxia medical university, The third batch of special talents launched in 2017 of Ningxia medical university (XT2017028), Undergraduate innovation and entrepreneurship project of Ningxia Hui Autonomous Region (S201910752013).

## Conflict of interests

None declared.

## Ethical approval and consent to participate

Informed consent was obtained from each participant. The animal experiments were performed in accordance with the guidelines of the Chinese Council on Animal Care.

## Supporting information


**Fig. S1.** Histograms showing a broad range of biochemical properties of the identified MTB‐CFPs. (A) molecular weight (Mw), (B) Isoelectronic point (pI), (C) grand average of hydropathy (GRAVY) distributions, and (D) number of TMD (trans‐membrane domain) proteins.

**Table S1.1** List of all identified MTB‐CFPs from *M. tuberculosis* H37Rv using LC‐MS/MS.
**Table S1.2** Discription of functional group of all identified MTB‐CFPs from *M. tuberculosis* H37Rv.

**Table S2.1** List of the first time identifications in MTB‐CFPs from *M. tuberculosis* H37Rv using LC‐MS/MS.
**Table S2.2** List of the second time identifications in MTB‐CFPs from *M. tuberculosis* H37Rv using LC‐MS/MS.
**Table S2.3** List of the third time identifions in MTB‐CFPs from *M. tuberculosis* H37Rv using LC‐MS/MS.

**Table S3.** The list of the sequences of primers for target genes PCR reaction.
