# Supplementary material for: emPAI‐assisted strategy enhances screening and assessment of Mycobacterium tuberculosis infection serological markers
Source: Microb Biotechnol. 2021 Jun 26;14(4):1827–38. doi: 10.1111/1751-7915.13829 (PMC8313264; doi:10.1111/1751-7915.13829)
Supplement: Supplementary file 1 — Fig. S1. Histograms showing a broad range of biochemical properties of the identified MTB‐CFPs. (A) molecular weight (Mw), (B) Isoelectronic point (pI), (C) grand average of hydropathy (GRAVY) distributions, and (D) number of TMD (trans‐membrane domain) proteins. [file MBT2-14-1827-s003.docx]

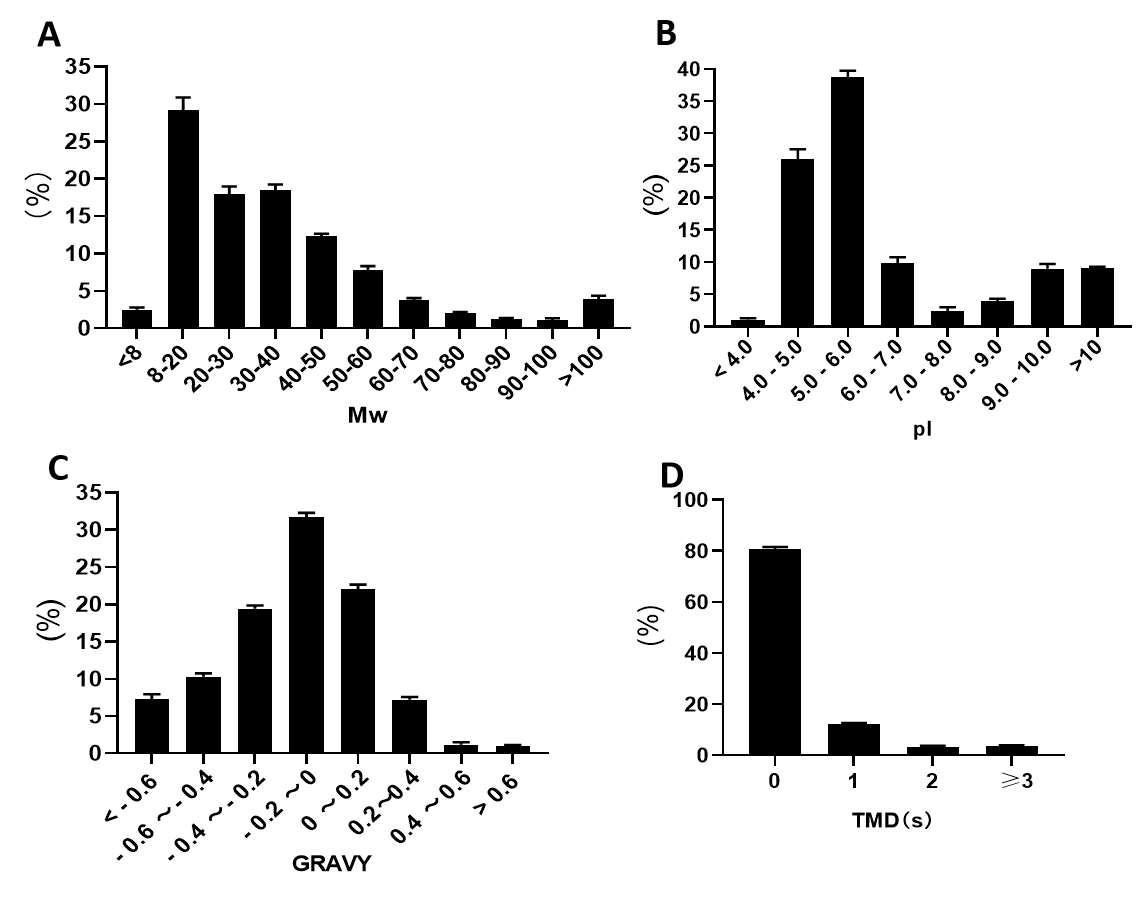


Fig.S1. Histograms showing a broad range of biochemical properties of the identified MTB-CFPs. (A) molecular weight (Mw), (B) Isoelectronic point (pI), (C) grand average of hydropathy (GRAVY) distributions, and (D) number of TMD (trans-membrane domain) proteins.
